# Prevalence and Risk Factors of Metabolic Syndrome: A Prospective Study on Cardiovascular Health

**DOI:** 10.3390/medicina59101711

**Published:** 2023-09-25

**Authors:** Marius Rus, Simina Crisan, Felicia Liana Andronie-Cioara, Mirela Indries, Paula Marian, Oana Lilliana Pobirci, Adriana Ioana Ardelean

**Affiliations:** 1 Department of Medical Disciplines, Faculty of Medicine and Pharmacy, University of Oradea, 410073 Oradea, Romania; paula.marian85@gmail.com; 2 Faculty of Medicine and Pharmacy, University of Oradea, 410073 Oradea, Romania; felicia_cioara@yahoo.com (F.L.A.-C.); mirela.indries@gmail.com (M.I.); oanap_30@yahoo.com (O.L.P.); adriana_toadere@yahoo.com (A.I.A.); 3 University of Oradea, 410073 Oradea, Romania; 4 Cardiology Department, “Victor Babes” University of Medicine and Pharmacy, 2 Eftimie Murgu Sq., 300041 Timisoara, Romania; urseanusimina@yahoo.com; 5 Institute of Cardiovascular Diseases Timisoara, 13A Gheorghe Adam Street, 300310 Timisoara, Romania; 6 Research Center of the Institute of Cardiovascular Diseases Timisoara, 13A Gheorghe Adam Street, 300310 Timisoara, Romania; 7 Department of Psycho Neuroscience and Recovery, Faculty of Medicine and Pharmacy, University of Oradea, 410073 Oradea, Romania; 8 Department of Preclinical Disciplines, Faculty of Medicine and Pharmacy, University of Oradea, 410073 Oradea, Romania

**Keywords:** metabolic syndrome, rising prevalence, cardiovascular disease, risk factors, obesity, high blood pressure, dietary habits, preventive strategies, management

## Abstract

*Background and objectives*: This article highlights the relationship between metabolic syndrome and cardiovascular disease, providing a comprehensive overview of its risk factors and prevalence. Metabolic syndrome, characterized by a cluster of interconnected risk factors, significantly increases the risk of developing cardiovascular disease and type II diabetes. *Materials and methods*: This study, conducted over a one-year period, involved 117 patients aged between 30 and 79 years old. Various parameters were analyzed, such as gender, age, education level, provenance from urban or rural environment, smoking, alcohol consumption, dietary aspects, physical activity, and their contribution to the appearance of metabolic syndrome. Central adiposity and high blood pressure emerged as prominent elements of the condition. *Results:* The findings underscore the importance of a healthy lifestyle in the prevention and management of metabolic syndrome. Encouraging regular physical activity, maintaining a balanced diet, rich in fresh vegetables and fruits, and avoiding harmful behaviors, such as smoking or alcohol consumption, are essential in reducing the risk of metabolic syndrome and its associated cardiovascular complications. *Conclusions*: The study highlights the need for public health initiatives, as well as individualized preventive strategies to combat the rising prevalence of metabolic syndrome. Through promoting awareness of its risk factors and implementing effective interventions, healthcare professionals can contribute to better cardiovascular health worldwide. Further research in this area will continue to enhance our understanding of metabolic syndrome and refine preventive and therapeutic approaches for its management.

## 1. Introduction

The main cause of mortality and morbidity of the adults and elderly worldwide is cardiovascular disease, primarily due to coronary and non-coronary atherosclerosis. The Framingham Heart Study (FHS), a multigenerational study initiated in 1948, had a major contribution in understanding the causes of coronary disease, stroke, and other cardiovascular pathologies, as well as their prevention strategies. The Framingham study defined these causes as risk factors for heart disease. Among all these factors, FHS emphasized the major independent elements such as smoking, arterial hypertension, high total cholesterol, and LDL-cholesterol (LDL-c), low HDL-cholesterol (HDL-c), diabetes mellitus type II, age, obesity, and a sedentary lifestyle [1]. In the forthcoming years, substantial research was concentrated on the clinical evaluation of these risk factors. However, as time passed, progresses and new discoveries shifted the attention to other screening and diagnostic methods of cardiovascular diseases, such as adipose tissues biomarkers (leptin, resistin, adiponectin), proinflammatory status biomarkers (high sensitive C reactive protein—hsCRP), prothrombotic status biomarkers (fibrinogen, plasminogen activator inhibitor-1—PAI-1), abnormal fat tissue distribution (visceral fat, hepatic steatosis), insulin resistance (IR) and hyperinsulinemia (HI) [1,2]. Consequently, metabolic syndrome was defined as a cluster of clinical and biological elements that increase the risk to develop a cardiovascular disease [2]. In population-based studies, it was revealed that individuals diagnosed with metabolic syndrome presented a twofold elevation in the risk of cardiovascular events. Moreover, for non-diabetic patients, the risk of developing type II diabetes increased up to five times [2,3,4].

The first definition of metabolic syndrome was formulated in 1999, by the World Health Organization (WHO), emphasizing hyperglycemia and insulin resistance as the main criteria for diagnosing the condition [5]. The NCEP/ATP III (National Cholesterol Education Program/Adult Treatment Panel III) report, published in 2001, modifies the definition of metabolic syndrome, suggesting that three or more of the following criteria are diagnostic criteria for metabolic syndrome: (1) waist circumference > 102 cm in men and >88 cm in women, (2) concentration of triglycerides ≥150 mg/dL (1.7 mmol/L), (3) concentration of HDL-c < 40 mg/dL (1.03 mmol/L) in men and <50 mg/dL (1.29 mmol/L) in women, (4) systolic blood pressure ≥ 130 mm Hg or a diastolic blood pressure ≥ 85 mm Hg, and (5) fasting glucose ≥ 110 mg/dL (≥5.6 mmol/L) [3]. However, the International Diabetes Federation (IDF) released new criteria four years later, highlighting central adiposity as a mandatory element for the condition, plus two or more factors: (1) raised levels of triglycerides ≥ 150 mg/dL (1.7 mmol/L) or specific treatment for this lipid abnormality, (2) low concentration of HDL-c < 40 mg/dL (1.03 mmol/L) in men and <50 mg/dL (1.29 mmol/L) in women or specific treatment, (3) high systolic blood pressure ≥ 130 mm Hg or high diastolic blood pressure ≥ 85 mm Hg, or treatment of previously diagnosed hypertension, and (4) increased concentration of fasting glucose ≥ 100 mg/dL (5.6 mmol/L) or previously diagnosed type II diabetes [2,3]. In 2009, the American Heart Association (AHA) and the National Heart, Lung, and Blood Institute (NHLBI), together with the IDF, attempted to unify all these components. As a result, waist circumference was no longer a mandatory element of the criteria used to define the metabolic syndrome [6].

The wide variety of interpretations led to substantial confusion regarding the concept of metabolic syndrome [7]. Thus, the metabolic syndrome is a pathological cluster of interconnected metabolic risk factors that directly increase the risk of developing atherosclerotic coronary disease and type II diabetes [8].

The underlying causes of metabolic syndrome are complex and involve a combination of genetic, environmental, and lifestyle elements, such as poor diet, sedentary behavior, and high body mass. The syndrome’s prevalence is increasing globally, some causes being the higher rates of obesity, especially abdominal obesity, and type II diabetes. Increased insulin resistance, along with old age and central adiposity represent the main pathophysiological mechanisms which explain the increasing of metabolic syndrome prevalence [2,9].

The management of metabolic syndrome involves lifestyle changes. Weight loss, proper nutrition and adequate physical activity are the fundamental elements of treatment. Therapeutic strategies for correcting modifiable risk factors such as high blood pressure or dyslipidemia, should be applied according to existing guidelines. Early detection and intervention are also crucial in lowering the risk of associated cardiovascular complications [2,10].

This study aimed to establish the correlation between environmental elements, poor lifestyle choices, and the risk of developing metabolic syndrome. We systematically analyzed the relation between this condition and gender, background, education level, smoker/non-smoker status, physical activity, and diet. Identifying a specific diet, or certain nutrients, which could reduce the risk of developing metabolic syndrome, would enhance existing cardiovascular prevention strategies and the prognosis of metabolic syndrome.

## 2. Materials and Method

A total of 117 patients were included in the study (53 men, and 64 women), aged between 30 and 79 years old. It was a prospective study, conducted between January 2022–February 2022. The study participants were selected among the patients who presented themselves for a cardiological consultation, being addressed by the general practitioner.

The study protocol was approved by the ethics committee of Bihor County Clinical Emergency Hospital Romania. Paraclinical investigations, as well as clinical examinations, were performed for every patient. The main parameters of interest were evaluated at the inclusion (T1): total cholesterol level (TC), blood pressure (BP), triglycerides values (TG), HDL-c, and plasma fasting glucose (PFG). All of the measurements were repeated after 6 and 12 months, thus creating three time points (T1, T2, T3) for comparing the results. Additionally, all the patients filled out a questionnaire regarding their socioeconomic status (age, level of education, and occupation), dietary habits, smoking and alcohol consumption, and physical activity.

We analyzed the patients’ dietary habits, considering the number of meals per day (≥3/day vs. <3/day) and the preference for certain types of food.

Regarding the consumption of lipids, the patients declared the following:

- No consumption or low consumption;
- Consumption in high quantities.

Concerning meat consumption, the patients were divided into three groups:

- No consumption;
- Red meat preference—beef, pork, lamb (rich in saturated fatty acids);
- White meat preference—herring, salmon, mackerel, tuna, sardine (rich in unsaturated fatty acids from the omega-3 group) and bird meat (rich in omega-6 fatty acids).

Based on fresh fruits and vegetables consumption (omega-3, omega-6 and omega-9 acid sources) study participants were classified as follows:

- Non-consumers;
- Daily or weekly consumers.

Carbonated drink consumers were divided into two groups:

- Non-consumers;
- Regular consumers.

Concurrently, the salt intake was quantified, and the patients were divided as follows:

- No salt consumption, used sparingly or dietary salt;
- A diet characterized by a high salt intake.

The patients were grouped into four categories based on smoking or alcohol consumption:

- Non-smoker/no alcohol usage;
- Smoker/alcohol consumers.

Regarding physical activity, we considered individuals as physically active if trainings of moderate intensity were practiced at least 5 times a week, such as 30–60 min fast walks.

Furthermore, patients included in this study were classified into 3 categories based on their education level:

- Higher education level—postgraduate studies, undergraduate studies, vocational school;
- Medium education level—high school;
- Lower education level—primary education.

In this study, cardiovascular prevention strategies were applied. Apart from pharmaceutical therapies, the participants were also given advice meant to correct an unbalanced lifestyle, which elevates the risk of developing metabolic syndrome. The smokers were included in smoking-cessation programs, while withdrawal symptomatology was diminished with the help of nicotine gum, sprays, or patches. On the recommendation of the specialist physician, the participants were included in individualized sport programs and they also received recommendations for a healthy diet.

To establish the diagnosis of metabolic syndrome, we used the IDF/AHA/NHLBI (2009) consensus definition [3]. The exclusion criteria included the following categories of patients:

- Individuals with insufficient data, especially regarding serum levels of PFG, TG, HDL-c, blood pressure, body mass index (BMI);
- Patients diagnosed with infectious diseases, including hepatitis B or C, liver or renal failure, hepatic cirrhosis, oncological diseases;
- Patients deceased during the study.

The statistical analysis of the data was performed with Statistica 8.0. The statistic performed was a *t*-test for numeric variables, chi-square test and cross-tabulation for categorical variables, and univariate analysis and multiple regression for independent predictors of metabolic syndrome.

The statistically significant value considered was *p* ˂ 0.05.

## 3. Results

The study was conducted between January 2022 and February 2023. The main characteristics of the study participants are presented in Table 1.

**Figure 1:**
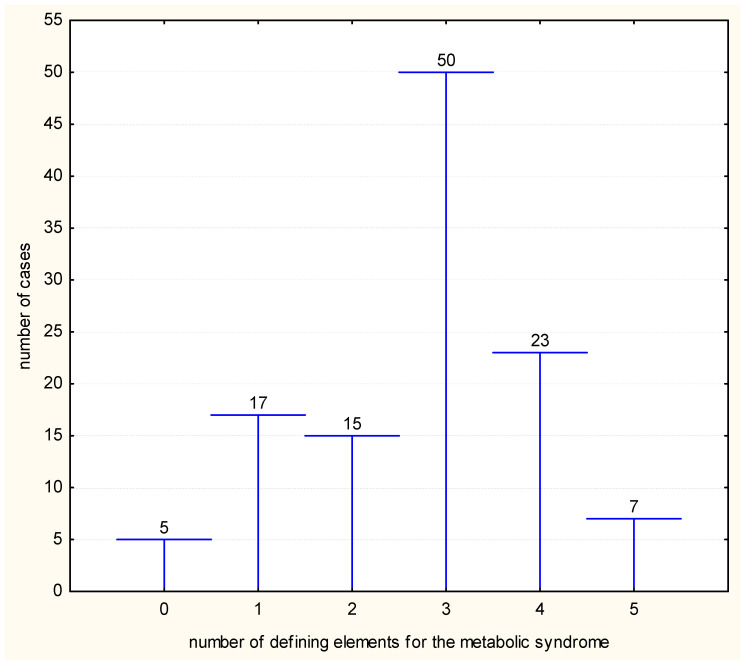
Distribution of cases according to number of defining elements for metabolic syndrome.

At the initial evaluation (T1), our results indicated that the incidence of the metabolic syndrome reached a value of 68.37% in the examined population. The analysis of the patients according to the presence of the defining elements for the metabolic syndrome identified five patients without any element (4.27%), 17 patients that had one element (14.52%), 15 patients with three elements (12.82%), 50 patients with three elements (42.73%), 23 patients with four elements (19.65%) and 7 patients (5.98%) that presented all of the five elements (*p* ˂ 0.0001) (Figure 1).

Thus, 82 patients presented a minimum of three elements of the metabolic syndrome out of five, while 37 individuals presented two, one, or no factors. Central adiposity was the most prevalent component, identified in 76.92% of the participants. The second most common factor was arterial hypertension, with an incidence of 70.08%, while high fasting glucose was reported only in 33.33% of the cases.

The main predictors for metabolic syndrome at T1 were lipid intake and lack of physical activity (*p* ˂ 0.05).

Furthermore, the incidence of metabolic syndrome was assessed based on the gender distribution: females 52.5% (*n* = 42) and 47.5% males (*n* = 38), *p* = 0.73.

The patients were divided in five groups based on the age at the inclusion (Table 2). The metabolic syndrome was more prevalent in individuals over 60 years old (59%).

It was observed that the gender distribution of metabolic syndrome was directly proportional with age, with no significant differences between men and women regarding the higher prevalence of the metabolic syndrome in individuals over 60 years old (*p* = 0.13). The increasing incidence of metabolic syndrome with age was present in both men and women (correlation coefficient r = 0.24, *p* = 0.009).

The distribution of the elements that define the metabolic syndrome by age category is shown in Table 3.

A significant variation between groups was noticed regarding the presence of arterial hypertension, central adiposity and high fasting glucose. Arterial hypertension tended to be more prevalent after 50 years of age, central adiposity was more prevalent in the fourth and sixth decade, and high fasting glucose in individuals aged between 60 and 69 years old.

A higher education level (vocational training, university, postgraduate studies—Table 4) was present in 30.7% of patients. The metabolic syndrome was more prevalent in patients with low- or medium-level studies, with a significant difference between the last two categories (48% vs. 21.3%, *p* = 0.0008).

Regarding environment, our data suggested that a rural background could be a predisposing factor of metabolic syndrome (78.6% versus 51.10%, *p* = 0.001).

**Figure 2:**
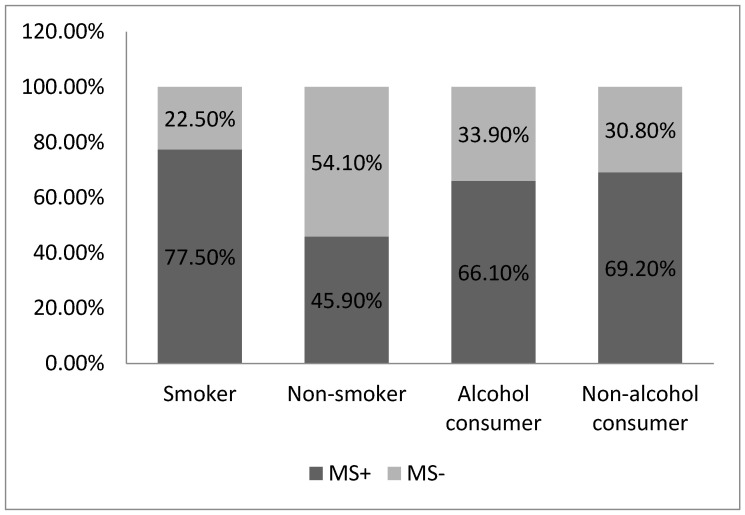
The incidence of metabolic syndrome among smokers and alcohol consumers (MS”+” = patients with metabolic syndrome; MS”-“= patients without metabolic syndrome).

Considering the correlation between smoking and metabolic syndrome, we noted that a smoker status represented a considerable risk factor for the condition. Regarding alcohol consumption, our data analyses did not find a major correlation between this parameter and the presence of metabolic syndrome. An amount of 66.10% of study participants were alcohol consumers, while 69.2% were non- alcohol consumers (*p* = 0.12) (Figure 2).

Furthermore, in terms of alcohol beverages, the results revealed that patients with metabolic syndrome preferred beer (41%) and wine (33%) over hard liquors (23%).

The relationship between dietary habits and metabolic syndrome was also analyzed. The patients were investigated regarding the number of meals per day, lipid intake (saturated or trans), meat consumption, salt intake, fresh vegetables and fruit intake and carbonated drink consumption.

It was observed that metabolic syndrome was more frequent in individuals that took under three meals per day: 30 out of 41 patients (73.2%), who ate only 2 meals per day, were identified with metabolic syndrome (*p* = 0.004). Moreover, 4 out of 5 patients (80%), who declared to only consume one meal/day, were also identified with the condition.

Other significant risk factors were the consumption of lipids, saturated and trans, and the preference for red meat. The exclusive intake of white meat was a protective factor against the syndrome (42.5% vs. 70.7%, r = 0.32, *p* = 0.032). Overall, a diet rich in meat was revealed to favor the appearance of metabolic syndrome, due to a higher percentage of consumed red meat.

The results of our study also suggested that the consumption of fresh fruits and vegetables exhibited a protective role against the onset of metabolic syndrome, even in individuals with unhealthy dietary habits, such as a high intake of salt or carbonated drinks (*p* = 0.036).

Finally, the correlation between physical activity and the incidence of metabolic syndrome was analyzed. A sedentary lifestyle was proven to be a considerable risk factor for the appearance of the condition.

At the 6-month visit (visit T2), the prevention recommendations were followed by 76 participants (64.95%). Nevertheless, we could still clearly observe a decreased incidence of metabolic syndrome among the studied population (*n* = 69.59%).

Regarding the frequency of the elements defining the metabolic syndrome, central obesity remained in the first place, identically to the start of the study, while the least commonly encountered factor was a low level of HDL-col. (Table 1). Regarding gender distribution in the second point of the study (T2), males maintained a higher risk in developing metabolic syndrome compared to women, but this was not significant (45.3% versus 54.7%, *p* = 0.92).

After 12 months, during the final visit (T3 visit), the prevalence of metabolic syndrome was still important but a slight decrease was noticed compared with the initial evaluation (74 patients, 63.2%).

**Figure 3:**
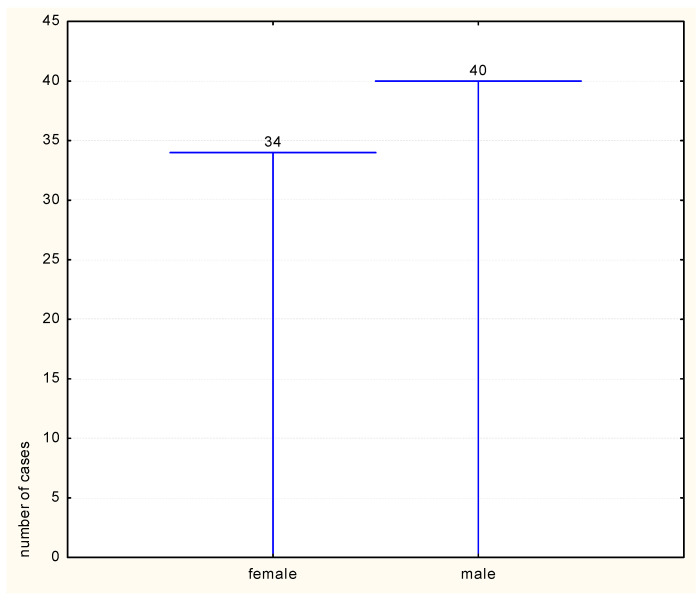
Metabolic syndrome distribution by gender.

Regarding the factors that define the metabolic syndrome, central adiposity manifested a progressive increase (54.70%), while increased fasting glucose was the poorly represented element (25.6%). The gender distribution of metabolic syndrome indicated a higher risk of the condition in men compared to women (Figure 3).

The higher prevalence of metabolic syndrome in individuals over 60 years old, noticed at the initial visit, was noticed at the final evaluation, in both men and women with a significant correlation with age (correlation coefficient = 0.44, *p* ˂ 0.0001).

The evolution of the prevalence of each element defining the metabolic syndrome was displayed over the course of the study. An improvement was highlighted in BP, TG and HDL-c levels. At the final visit central obesity and high fasting glucose were present in 54.70% of the studied population, and arterial hypertension in 68.9%.

A high value of the fasting glucose remained the least observed element of metabolic syndrome (25.6%). This observation was interesting since the individuals were predominantly obese and it was expected they had a secondary hyperinsulinemia, with a negative impact on glycemic values. Therefore, we analyzed the recorded predictors for glycemic values in obese patients, and the strongest predictor for normal glycemic values in obese patients was regular physical activity (*p* ˂ 0.0001).

## 4. Discussions

Metabolic syndrome is a public health concern, due to its high and continuously increasing prevalence and incidence, even gaining a pandemic trait in recent years [11]. The purpose of this study was to evaluate the risk factors involved in metabolic syndrome and emphasize their significant role in developing cardiovascular complications. We identified non-modifiable risk factors, such as gender and age, and modifiable elements, such as low educational levels, rural environmental provenience, smoking, a diet rich in lipids and poor in fresh vegetables and fruits, as well as a sedentary lifestyle.

The prevalence of metabolic syndrome over the course of the study (January 2022–February 2023) was 63.3%. Regarding gender distribution, our data suggested that men present a higher prevalence of the syndrome compared to women (52.5% versus 47.5% males, *p* = 0.73).

Our data highlighted that the prevalence of metabolic syndrome among men remained higher within the study, in T3, the discrepancies between genders becoming almost statistically insignificant. We concluded that men have a slightly higher risk of developing the syndrome, with numerous other studies, such as SYMFONIE or the SEPHAR study, supporting this claim [12,13,14,15,16].

However, while other papers claim that the prevalence of metabolic syndrome increases in women along with age, reaching a value equal to that of men, if not higher [17,18,19], in our study, both genders displayed a higher risk to develop metabolic syndrome related to age. The most affected age groups were aged between 60–69 years old and the 70–79-year-old group, categories where women had a higher risk to develop the disease. For the rest of the age categories, the incidence and prevalence continued to be higher among men.

Regarding each parameter that defines metabolic syndrome, central adiposity was the most frequent component, its prevalence decreasing in T2, most probably secondary to applying of the measures targeted to weight loss. The fasting glucose presented a similar course. Through pharmaceutical therapies, as well as dietary changes (regular meals, low salt intake, white meat consumption, fresh vegetables and fruits, zero consumption of soft drinks), the high blood pressure was regulated, while the lipidic profile improved. As a result, the prevalence of the other elements decreased. Arterial hypertension was controlled in most of the patients, secondary to pharmacological and non-pharmacological actions, while a high value of the fasting glycemia was the least-counted element.

At the final visit evaluation, central obesity remained the most prevalent component of the syndrome, while the prevalence of high blood pressure decreased (with 2%). Fasting glucose remained the least frequently encountered factor, considering it was the easiest to correct by diet, weight loss and specific medication. Blood pressure showed a fair reduction, our data reporting a value of 68.9%. As for the lipidic profile, TG increased from 34.18% in T2 to 38.5% in T3. One of the most impressive improvements was the correction of HDL-cholesterol values (from 64.95 at T1 to 42.73% at T3, *p* ˂ 0.0001).

These findings support preexisting data that suggest that metabolic syndrome increases the risk of cardiovascular disease onset, such as myocardial infarction, similar with the INTERHEART study observations [15]. In the ROMES study, however, stable angina was directly associated with the metabolic syndrome in more circumstances than acute coronary syndromes [14]. Another study discusses the correlation between the risk of heart failure (HF) and metabolic syndrome, arguing whether the condition increases HF risk independently [20]. Atrial fibrillation (AF), it is still obscure if the condition is due to the syndrome as a whole, or the risks of its individual elements [21]. Other works suggest that certain components of the metabolic syndrome, such as a high waist circumference, increase the risk of several organ dysfunctions, especially in women [12,21,22,23].

Our data suggest that rural environment of origin as well as a low education level could have a negative impact on metabolic syndrome prevalence.

Both education, as well as background, are two important risk factors, discussed in several specialty works, such as the PURE study [15,17,24,25]. There was a significant variation between lower educated people, and patients with studies of higher levels suffering from the syndrome, whilst a considerable discrepancy could be observed between the rural and urban population. Thus, we can conclude that the incidence and prevalence of the metabolic syndrome varies by socioeconomic position, but more epidemiological studies are needed to further research this theory.

Two major factors that influenced the appearance of metabolic syndrome were smoking and poor dietary habits. Metabolic syndrome was more frequent in smokers compared to non-smokers. An unbalanced diet, with less than three meals per day, proved to be an important risk factor. A high intake of saturated fats, originating from red meat, and trans fats, mostly found in junk food, increased the risk of developing the syndrome, with exclusive white meat consumption proving to be a protective factor against the condition.

Another protective factor against the metabolic syndrome onset was the intake of fresh vegetables and fruits, even though the difference in the prevalence was almost insignificant. Despite all this evidence, overall meat consumption enhanced the risk of developing the metabolic syndrome, although this fact could be attributed to higher intake of red meat, compared to white meat.

Other patterns related to diet, which may increase the risk of metabolic syndrome, are high salt intake and considerable consumption of carbonated drinks. Significantly more patients who consumed augmented quantities of salt per day suffered from metabolic syndrome. Additionally, the prevalence of metabolic syndrome was higher among individuals who regularly consumed soft drinks. The EPIC (European Prospective Investigation into Cancer and Nutrition) cohort brings forth evidence that both artificially and sugar-sweetened soft drinks are associated with all-cause mortality [10,26].

Thus, diet plays a vital role in the pathophysiology of the metabolic syndrome. There is straightforward evidence to support that healthy dietary habits, such as the Mediterranean Diet (MedDiet), which not only facilitates weight loss, but also regulates plasma glucose and LDLc and TG levels, decreasing inflammation and blood pressure, further lowers the risk of metabolic syndrome [27,28,29,30]. The PREDIMED study supports the inverse association between cardiovascular risk and adherence to the Mediterranean diet [28]. Other prevention strategies, such as the Dietary Approach to Stop Hypertension (DASH), or country-specific dietary guidelines, such as Healthy Eating Indices (HEI), serve as alternatives to the MedDiet, being consistently associated with a reduced risk of metabolic syndrome [28,31,32,33,34].

In this study, there was no direct association between alcohol consumption and metabolic syndrome; therefore, it did not count as a risk factor. Metabolic syndrome was more frequent in individuals who did not consume alcohol at all, compared to the ones who drank moderate quantities. The type of beverage also played a key role in this theory, the majority of patients suffering from the condition preferring wine or beer, while a small part consumed solely hard liquors. However, the concept that moderate alcohol consumption is associated with lower cardiovascular risk, is not supported by several works, which suggest that lower risk for cardiovascular disease outcomes can actually be found in abstainers. These studies bring forth the theory that any amount of alcohol increases blood pressure and the BMI, thus further emphasizing the risk for metabolic syndrome overall [35,36,37,38].

Concerning physical activity, a sedentary lifestyle played a key role in the pathogenesis of the syndrome. Physical activity should be prescribed and tailored to each individual, according to the guidelines. Sports engagement is long associated with favorable metabolic health results in numerous studies, while sedentary behavior is associated with a greater risk for cardiovascular disease [38,39,40,41,42].

Another important observation of the study was that obese patients who practice regular physical exercises were less likely to develop diabetes, a fact already known and proven once again by the results of our study.

## 5. Conclusions

The study confirmed that gender and age play significant roles in the prevalence of metabolic syndrome. Men generally had a higher risk compared to women, but both genders exhibit an increased risk with advancing age. Healthcare practitioners should consider these factors when designing preventive strategies and interventions.

The impact of socioeconomic factors on the development of metabolic syndrome was highlighted. Unhealthy lifestyle habits including smoking, harmful dietary habits, and sedentary behavior are major risk factors for metabolic syndrome and require targeted interventions. Encouraging individuals to adopt and maintain healthier dietary patterns, such as the MedDiet, DASH, or country-specific dietary guidelines, can help in preventing and managing the condition.

Regular physical activity remains an important protective factor against metabolic syndrome. Sedentary behavior was strongly associated with an increased risk. Engaging in regular exercise and moderate-intensity activities can help reduce the likelihood of developing the condition.

The study underscores the importance of early detection and intervention in managing metabolic syndrome and its associated cardiovascular complications. Implementing lifestyle modifications, such as weight loss, proper nutrition, and increased physical activity, can significantly reduce the risk of the syndrome, and improve overall cardiovascular health.

Healthcare professionals should find and apply preventive strategies based on individual risk profiles, considering factors such as age, gender, socioeconomic status, and lifestyle choices. Personalized interventions are essential in addressing the diverse factors contributing to the metabolic syndrome. The results emphasize the need for further research in understanding the metabolic syndrome and its risk factors. Additionally, public health initiatives should focus on raising awareness about metabolic syndrome, its consequences, and the benefits of adopting a healthy lifestyle to prevent its occurrence.

## Figures and Tables

**Table 1:** Characteristics of the patients included in the study.

Age	57.74 ± 13.68 Years			
Sex	Female *n* = 64 (54.70%)		Male *n* = 53 (45.29%)	
	T1, n.%	T2, n.%		T3, n.%
Metabolic syndrome	80 (68.37%), *p* = 0.0001	69 (59%), *p* = 0.06		74 (63.2%), *p* = 0.005
Central adiposity	90 (76.92%), *p* ˂ 0.0001	61 (52.1%), *p* = 0.71		64 (54.70%), *p* = 0.35
High blood pressure, mm Hg	82 (70.08%), *p* ˂ 0.001	48 (41.02%), *p* = 0.06		51 (43.58%), *p* = 0.19
High triglycerides level, mg/dL	72 (61.53%), *p* = 0.016	40 (34.18%), *p* = 0.0009		45 (38.5%), *p* = 0.01
Low HDL-cholesterol, mg/dL	76 (64.95%), *p* = 0.0017	58 (49.57%), *p* = 0.99		50 (42.73%), *p* = 0.13
High fasting glucose, mg/dL	39 (33.33%), *p* = 0.0004	29 (24.7%), *p* ˂ 0.001		30 (25.6%), *p* ˂ 0.001
Number of meals/day	1 to 6, *p* ˂ 0.0001	2 to 6, *p* = 0.005		3 to 6, *p* = 0.04
Alcohol consumption	44, (37.60%), *p* = 0.0122			
Smoking	66, (54.45%), *p* = 0.19			
Lipids intake	81, (69.23%), *p* ˂ 0.0001			
Carbonated drink intake	58,(49.57%), *p* ˂ 0.001			
Salt intake	85, (72.64%), *p* ˂ 0.0001			
Meat intake	No intake 21, (17.94%)		*p* ˂ 0.001	
	White meat 79, (67.52%)			
	Red meat 49, (41.88%)			
Fruits and vegetables intake	*n* = 74, (63.24%), *p* = 0.005			
Physical activity	*n* = 45, (38.48%), *p* = 0.016			

**Table 2:** The correlation between age and prevalence of metabolic syndrome (*n* = number of patients, %).

Age Category	Metabolic Syndrome (T1) *n*, (% of Total Number of Patients)
30–39 years (*n* = 15)	7 (8.8%)
40–49 years (*n* = 18)	9 (11.3%)
50–59 years (*n* = 25)	17 (21.2%)
60–69 years (*n* = 30)	25 (31.2%)
>70 years (*n* = 29)	22 (27.5%)
	*p* = 0.003

**Table 3:** Distribution of the elements that define metabolic syndrome by age.

Age Category	Central Adiposity	High Blood Pressure, mm Hg	High Triglycerides Level, mg/dL	Low HDL-Cholesterol, mg/dL	High Fasting Glucose, mg/dL
30–39 years (*n* = 15)	9 (60%)	8 (53.33%)	8 (53.33%)	4 (26.67%)	2 (5.1%)
40–49 years (*n* = 18)	16 (88.89%)	10 (55.56%)	10 (55.56%)	6 (33.33%)	7 (17.9%)
50–59 years (*n* = 25)	18 (72%)	21 (84%)	16 (64.00%)	8 (32%)	6 (15.4%)
60–69 years (*n* = 30)	25 (83.33%)	22 (73.33%)	18 (60%)	12 (40%)	15 (38.5%)
>70 years (*n* = 29)	22 (75.86%)	21 (72.41%)	20 (68.97%)	11 (37.93%)	9 (23.1%)
	*p* = 0.08	*p* = 0.02	*p* = 0.29	*p* = 0.24	*p* = 0.02

**Table 4:** Patient distribution based on education level.

Completed Studies	Number of Patients (*n*)	Percentage (%)
Primary school	10	8.5%
Middle school	15	12.8%
Trade school	7	6%
High school	49	42%
Vocational training	8	6.8%
University	26	22.2%
Postgraduate studies	2	1.7%
Total	117	100%

## Data Availability

Not applicable.
